# Stability of Autistic Traits from 5 to 8 Years of Age Among Children in the General Population

**DOI:** 10.1007/s10803-018-3770-z

**Published:** 2018-10-05

**Authors:** Hideyuki Haraguchi, Andrew Stickley, Aya Saito, Hidetoshi Takahashi, Yoko Kamio

**Affiliations:** 1 Department of Preventive Intervention for Psychiatric Disorders, National Institute of Mental Health, National Center of Neurology and Psychiatry (NCNP), 4-1-1 Ogawahigashi, Kodaira, Tokyo 187-8553 Japan; 2 The Stockholm Center for Health and Social Change (SCOHOST), Södertörn University, 141 89 Huddinge, Sweden; 3 Institute for Educational and Human Development, Ochanomizu University, 2-1-1 Ohtsuka, Bunkyo-ku, Tokyo, 112-8610 Japan

**Keywords:** Autistic traits, Stability, Preschool children, Social Responsiveness Scale

## Abstract

Little is known about the across time stability of autistic traits during the transition period from preschool to school age in the general population. The current study compared autistic traits assessed by a mother-reported quantitative measure, the Social Responsiveness Scale, at age 5 and 8 years and examined the intraclass correlation coefficients of scores across the period for 168 Japanese community-based children. Results showed that total and two subdomain-related autistic trait scores remained primarily stable in males and females. This stability was observed for both children with higher and lower autistic traits scores with a possible sex-specific pattern. Our findings suggest that autistic traits in the general population can be reliably assessed using quantitative measures for this age period.

Autism spectrum disorder (ASD) is a lifelong neurodevelopmental condition characterized by persistent deficits in reciprocal Social Communication and Interaction (SCI) across multiple contexts, and by the presence of restricted, repetitive patterns of behavior and interests (American Psychiatric Association 2013). Several research studies have shown that an ASD diagnosis is remarkably stable across time in clinic-referred and community child samples (Chawarska et al. 2009; Guthrie et al. 2013; Kočovská et al. 2013; Van Daalen et al. 2009). However, most children with ASD are not diagnosed until 4 years of age (Pringle et al. 2012) and even though early identification has been improved, many families continue to experience a stressful “diagnostic odyssey” (Zwaigenbaum et al. 2015). Indeed, a recent epidemiological study of 7–12-year-old South Korean community children revealed that many children without a history of psychiatric or psychological service use in regular schools met the criteria for a diagnosis of ASD (Kim et al. 2011).

There is extensive evidence which shows that quantitative autistic traits are continuously distributed even in the general population (reviewed in Constantino and Charman 2016). Assessing autistic traits in children is important because of the potential mental health implications it can have; children with higher autistic traits not only have educational needs but also mental health needs that must be addressed for their better school adaptation. In particular, previous epidemiological research demonstrated that higher autistic traits were associated with functional impairment such as peer problems and emotional and conduct problems in schoolchildren (Moriwaki and Kamio 2013; Skuse et al. 2009). Importantly, there is also some evidence that the negative effects of these traits may stretch across time. For example, a recent longitudinal community-based study showed that emotional symptoms and peer problems in schoolchildren aged 7 years were predicted by higher autistic traits assessed at age 5 (Saito et al. 2017). However, to the best of our knowledge, as yet, no study has examined whether autistic traits in the general population remain stable during the transition period from preschool to elementary school. This is an important omission. During this transition period a drastic change occurs in the daily environments of children, which might impact on functioning, especially of children with ASD or higher autistic traits even at a subthreshold diagnostic level. Given this, if autistic traits are stable during this time period it will be beneficial for school personnel/mental health professionals to identify children with higher autistic traits prior to when they start school, so that they can spot any behavioral changes occurring in these children and manage them appropriately without delay.

A previous study on the developmental trajectories of symptom severity in preschool children diagnosed with ASD revealed that severity appeared to be primarily stable, with approximately 11% of the sample showing a marked decrease in symptom severity (Szatmari et al. 2015). However, less is known about stability/change in autistic traits in non-clinical children over time. Several studies that have been undertaken have reported that autistic traits are stable in the general population. For example, Constantino et al. (2009) showed that autistic traits, as measured by the mother-reported Social Responsiveness Scale (SRS; Constantino and Gruber 2005), were highly stable across a 5-year period in male–male twin pairs with a mean age of 11.6 years, although there was a modest, albeit statistically significant, improvement in the mean SRS score. Robinson et al. (2011) investigated the developmental course of autistic traits as measured by the parent-reported Social and Communication Disorders Checklist (Skuse et al. 2005) at ages 7, 10, and 13 using data from the Avon Longitudinal Study of Parents and Children. They found that autistic traits were highly stable in both the general population and higher-scoring children. By sex, males showed a slight, but statistically significant improvement, while there was no mean change for females. Holmboe et al. (2014) used the parent-reported Childhood Autism Spectrum Test (CAST; Scott et al. 2002) to examine the longitudinal correlation of autistic traits across 8–12 years of age for children from the Twins Early Development Study. They found that there was a slight, but nonetheless statistically significant, decrease in CAST scores while intra-individual variation in autistic traits was highly preserved across the study period for both males and females. Each of the three CAST subscales [social impairments, communication impairments, and restricted and repetitive behaviors (RRBs) and interests] also exhibited a high degree of stability. Whitehouse et al. (2011) investigated the long-term stability of autistic traits from age 2 to 19 years in the general population. In their study, autistic traits at age 2 were assessed using the Pervasive Developmental Problems (PDPs) subscale from the Child Behavior Checklist for Ages 1.5–5 (Achenbach et al. 1987), while in early adulthood autistic traits were assessed with the self-reported Autism-Spectrum Quotient (AQ; Baron-Cohen et al. 2001). Items from each scale were further divided into two subscales corresponding to social and non-social autistic traits. For males, total and social autistic trait scores at age 2 were modestly but significantly correlated with the corresponding trait scores at age 19, whereas there were no significant longitudinal correlations for non-social autistic traits. For females, there were no significant correlations for total or subscale autistic traits.

These previous findings suggest that autistic traits may be stable during middle childhood for both sexes, with a slight sex difference. For males, a slight but significant improvement during middle childhood has been consistently reported (Constantino et al. 2009; Holmboe et al. 2014; Robinson et al. 2011), while the evidence suggests that intra-individual variation is preserved (Holmboe et al. 2014). For females, no mean change (Robinson et al. 2011) or a slight but significant improvement has been observed while intra-individual variation is also preserved (Holmboe et al. 2014). Against this background, the current study had several aims. First, to examine the degree of stability/change in autistic traits during the transition period from preschool to elementary school in a cohort of community children. Second, to determine the level of across-time stability/change in each of the SCI and RRB subdomains. Third, to explore whether there are sex-specific patterns regarding the stability/change in autistic traits during this transition period. Finally, we also aimed to examine whether the stability of autistic traits differs for children with a higher level of autistic traits compared to children with a lower level of autistic traits. If autistic traits are similarly stable for both of these subpopulations, it will support the notion that quantitative autistic traits can be reliably assessed for all community children irrespective of symptom severity.

## Methods

### Participants and Procedure

**Fig. 1:**
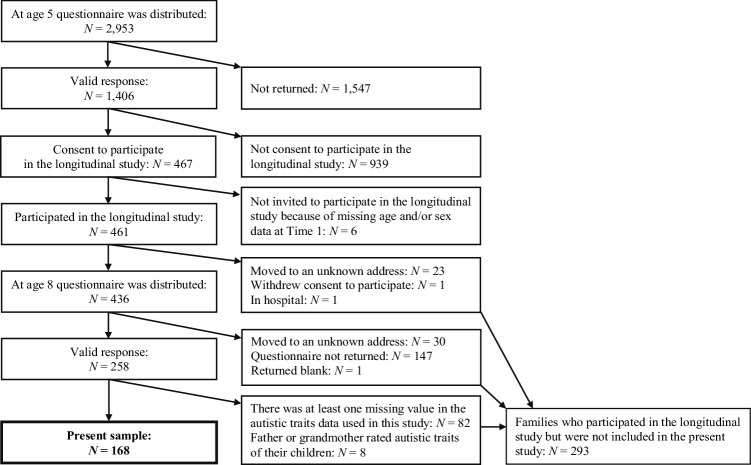
Flow diagram of the study participants

Participants in this study comprise a subsample of an ongoing longitudinal study, the Tama Children’s Survey (TCS). In 2011, the TCS was started to explore autistic symptom distribution and functional outcomes in childhood by targeting a cohort of community children (*N* = 461) living in the Tama District of Tokyo, Japan, that has been previously described elsewhere (Kamio et al. 2014; Saito et al. 2017). This report concerns the subsample whose prospective data sets at age 5 and 8 were complete, including information on sociodemographic status (SES). A flow diagram of the participants is presented in Fig. 1.

In 2011, with local government assistance, we invited all kindergartens and nursery schools (*N* = 78) located in two cities in the western suburbs (Tama) of Tokyo to participate in the TCS, and obtained agreement from 64 institutions. In March 2012 (at age 5 years), a questionnaire asking about autistic traits and sociodemographic factors accompanied by a cover letter explaining the purpose of the survey was distributed to 2953 families whose children were in classes for 5-year-olds via their teachers in these institutions. The questionnaire was returned voluntarily by 1406 parents (a response rate of 47.6%) among whom parents of 467 children gave written consent to participate in a subsequent longitudinal study. Six children were excluded because of missing age and/or sex data at age 5, which resulted in 461 children participating in the longitudinal study.

At age 8 years (September 2014), a questionnaire asking about autistic traits and other mental health problems was sent to these children apart from 25 children who could no longer participate for various reasons (23 had moved out of the area, 1 family withdrew consent, 1 child was hospitalized), with 258 being returned (a response rate of 59.2%). Of the 258 responses, we excluded 82 children with missing autistic traits data at age 5 and/or 8, and 8 children whose raters were not mothers at age 5 and/or 8. This resulted in data from 168 children (89 males) rated by the mother at both age 5 and 8 years being used in the current study.

#### Autistic Traits

In this study, autistic traits were assessed using the Japanese version of the SRS, a quantitative questionnaire of autistic symptomatology and traits for 4–18-year-olds that can be completed in about 15–20 min by parents (Constantino and Gruber 2005). Previous validation studies have demonstrated that the Japanese version of the SRS has good psychometric properties (Kamio et al. 2009, 2013a, b; Moriwaki et al. 2011). The SRS consists of 65 items that are scored using a 4-point scale with answer options extending from 0 (not true) to 3 (almost always true). The SRS total score ranges from 0 to 195, with higher scores indicating a greater degree of social impairment. Research has revealed that the SRS has a 2-factor structure (with 1 factor comprising 53 SCI items while the other comprises 12 RRB items) that corresponds to DSM-5 criteria (Frazier et al. 2014). The SCI score ranges from 0 to 159 and the RRB score ranges from 0 to 36. For this study, the mother-rated SRS total score and SCI and RRB subdomain scores were used in the analyses (Cronbach’s α = 0.93, 0.91, 0.84 at age 5, 0.95, 0.93, 0.86 at age 8 for 168 ratings, respectively).

T-scores converted from the SRS total scores based on a nationally representative standardization sample stratified by sex (Kamio et al. 2013b) were used to subdivide higher- and lower-scoring children following the original definition (Constantino and Gruber 2017).

#### Sociodemographic Factors

In addition to children’s sex, age, familial information such as fathers’ and mothers’ education level (years of schooling), employment status, and household income was also gathered.

#### Data Analyses

We initially examined if the current study sample was representative of the full sample at age 5 (*N* = 461) in terms of sex ratio, SES, or SRS scores. Next, we analyzed the across-time stability of autistic traits in two ways. First, to assess stability/change at the group level, a paired *t*-test was used to compare the mean SRS total, SCI, and RRB scores for all children between age 5 and age 8. A two-way analysis of variance (ANOVA) with repeated measures (sex × age) was also conducted using the SRS total, SCI, and RRB scores, respectively, in order to examine possible sex differences in stability. Second, to assess stability of autistic traits at the individual level, intraclass correlation coefficients (ICCs) for the SRS total, SCI, and RRB scores between age 5 and 8 years were calculated for the whole sample, and for males and females, respectively. ICCs were also calculated separately for the higher-scoring children whose T-score at age 5 was 60 or higher, and lower-scoring children whose T-score at age 5 was 59 or below. A T-score of 60 was chosen based on the original manual (Constantino and Gruber 2017). The strength of the association of the ICC values was classified as follows: below 0.20 slight, 0.21–0.40 fair, 0.41–0.60 moderate, 0.61–0.80 substantial, and 0.81–1.00 almost perfect (Landis and Koch 1977). A *p*-value of < 0.05 was considered statistically significant. All statistical analyses were performed with IBM SPSS Statistics, version 21.

## Results

### Sample Characteristics

Sample characteristics are shown in Table 1. The sex ratio of the present sample did not differ significantly from that of the 293 children that consented to participate in the longitudinal study but who were not included in this study [*χ*^2^(1) = 0.003, *p* = 0.956]. When these two groups were combined there was also no significant difference in the sex ratio between these 461 participants and the remaining non-participants (939 who did not provide consent and 6 with missing data who were not invited to join the longitudinal study) [*χ*^2^(1) = 1.098, *p* = 0.295, Fig. 1]. Thus, the sex ratio (89:79) of this sample was not different from that of the initial sample.

**Table 1:** Characteristics of the study participants (*N* = 168)

	*N* (%)	Mean (SD) range
Children		
Sex (male:female)	89:79	
At age 5 (years)		5.36 (0.29) 4.83–5.92
At age 8 (years)^a^		7.92 (0.29) 7.42–8.92
Parents		
Years of schooling (years)		
Father^b^		15.30 (2.27) 9–24
Mother		14.83 (1.75) 9–19
Employment		
Father^c^		
Employed	161 (98.8)	
Not employed	2 (1.2)	
Mother^d^		
Employed	65 (38.9)	
Not employed	102 (61.1)	
Household income^e^		
< 2 million JPY	4 (2.5)	
2 < 5 million JPY	30 (18.5)	
5 < 7 million JPY	47 (29.0)	
7 < 10 million JPY	46 (28.4)	
10 < 15 million JPY	28 (17.3)	
≥ 15 million JPY	7 (4.3)	

*JPY* Japanese Yen (in September 2012, 1 million yen = ca. US $ 12,800)

^a^Calculated for 167 participants

^b^Calculated for 164 participants

^c^Calculated for 163 participants

^d^Calculated for 167 participants

^e^Calculated for 162 participants

Regarding SES, when the final sample was compared with the 293 participants who were included in the study at age 5 but not at age 8, there were no significant differences in the fathers’ education level [*t*(440) = 0.348, *p* = 0.728], mothers’ education level [*t*(457) = 1.457, *p* = 0.146], the fathers’ rate of employment [*χ*^2^(1) = 0.299, *p* = 0.585], or household income [*χ*^2^(5) = 5.377, *p* = 0.372], although the mothers in the present sample were more likely to be full-time housewives than the mothers of the 293 children [*χ*^2^(1) = 4.160, *p* < 0.05]. When these 461 longitudinal study participants were compared with the non-participants, there were no significant differences in the fathers’ employment rate [*χ*^2^(1) = 0.049, *p* = 0.825], mothers’ employment rate [*χ*^2^(1) = 1.574, *p* = 0.210], household income [*χ*^2^(5) = 8.766, *p* = 0.119], the fathers’ education level [*t*(1308) = 0.812, *p* = 0.417] although the mothers’ education level was higher among participants [*t*(1367) = 2.698, *p* < 0.01]. Thus, the SES of the present sample seems similar to that of the initial sample (*N* = 1406) except that mothers have a higher educational level.

Comparing the present sample against data relating to the general Japanese population revealed that the mean years of fathers’ and mothers’ schooling (14.8 years) were longer than the national average (12.0 years) reported by the United Nations Development Program (2012), that the percentage of mothers who were full-time housewives in this sample (61%) was higher than the national average (47%) (Eto 2011), and that the proportion of low-income families (with under 2 million yen per year) (2.5%) was lower than that in a survey (5.8%) conducted by the Ministry of Health, Labour and Welfare in 2012 (Table 1).

### SRS Scores of the Study Participants

To examine if the SRS scores of the 168 participants in this study were representative of the full longitudinal baseline sample at age 5 (*N* = 461, Fig. 1), we compared the SRS scores of the 168 participants and the remaining 293 children who were not included in the study at age 8. At age 5, there were no significant differences in the SRS total scores, *t*(344) = − 0.854, *p* = 0.393; the SCI scores, *t*(344) = − 0.901, *p* = 0.368; and the RRB scores, *t*(344) = − 0.571, *p* = 0.568. Means, standard deviations and ranges for each measure are presented in Appendix. When examined separately by sex, there were also no significant differences in any SRS scores between the 168 participants and 293 non-participants. Means, standard deviations, ranges and *t*-test results for each measure are presented in Appendix. Among the 168 children, 26 had a T-score of 60 or higher while 142 children had a T-score of 59 or lower, indicating the score distribution of the present sample was similar to that of the Japanese normative sample (Kamio et al. 2013b).

### Change in Autistic Traits Mean Scores from Age 5 to 8 at the Group Level

The mean SRS total, SCI, and RRB scores at age 5 and 8 years are presented in Table 2. There were no statistically significant differences in any scores during the period for the whole sample [*t*(167) = 1.060, *p* = 0.291; *t*(167) = 0.829, *p* = 0.408; *t*(167) = 1.595, *p* = 0.113, respectively]. A two-way ANOVA revealed that the main effect of sex was significant only for the RRB scores [*F*(1, 166) = 5.374, *p* < 0.05] but not for the total [*F*(1, 166) = 2.484, *p* = 0.117] and SCI scores [*F*(1, 166) = 1.706, *p* = 0.193]. This indicates that males had significantly higher scores than females at both age 5 and 8 only for the RRB subdomain. Neither the main age effect nor the interaction between sex and age was significant [total: age *F*(1, 166) = 1.143, *p* = 0.287; interaction *F*(1, 166) = 0.053, *p* = 0.819. SCI: age *F*(1,166) = 0.717, *p* = 0.398; interaction *F*(1, 166) = 0.132, *p* = 0.716. RRB: age *F*(1, 166) = 2.460, *p* = 0.119; interaction *F*(1, 166) = 0.107, *p* = 0.744]. Thus, stability/change did not differ by sex.

**Table 2:** SRS scores at age 5 and 8 years

SRS raw scores	All children (*N* = 168)		*t*-Test	Male (*N* = 89)		Female (*N* = 79)		Two-way ANOVA		
	Age 5	Age 8		Age 5	Age 8	Age 5	Age 8	Sex	Age	Sex × Age
	Mean (SD)	Mean (SD)	*t*	Mean (SD)	Mean (SD)	Mean (SD)	Mean (SD)	*F*	*F*	*F*
	[Range]	[Range]		[Range]	[Range]	[Range]	[Range]			
Total	33.43 (19.32)	32.26 (21.90)	1.060	35.52 (20.52)	34.58 (23.27)	31.08 (17.70)	29.63 (20.06)	2.484	1.143	0.053
	[5–117]	[4–132]		[5–111]	[4–132]	[5–117]	[4–113]			
SCI	28.71 (15.36)	27.94 (17.68)	0.829	30.01 (16.26)	29.56 (18.73)	27.24 (14.24)	26.11 (16.34)	1.706	0.717	0.132
	[4–92]	[3–102]		[4–90]	[3–102]	[5–92]	[4–92]			
RRB	4.72 (4.65)	4.32 (4.91)	1.595	5.51 (4.91)	5.02 (5.26)	3.84 (4.19)	3.52 (4.37)	5.374*	2.460	0.107
	[0–25]	[0–32]		[0–21]	[0–32]	[0–25]	[0–23]			

*SRS* Social Responsiveness Scale, *SCI* Social Communication and Interaction, *RRB* Restricted Interests and Repetitive Behavior

*p < 0.05

### Stability of Autistic Traits from Age 5 to 8 at the Individual Level

The ICCs of the SRS total, SCI, and RRB scores for the whole sample are presented in Table 3. For all children and males, the ICCs of all scores were substantial (all *p*-values < 0.001). For females, the ICCs for the SRS total and SCI scores were similarly substantial while the ICC for the RRB score was almost perfect (all *p*-values < 0.001).

**Table 3:** Intraclass correlation coefficients of the SRS scores at age 5 and 8 years

Whole group	All children (*N* = 168)	Male (*N* = 89)	Female (*N* = 79)
SRS total	0.759***	0.750***	0.767***
SCI	0.738***	0.735***	0.738***
RRB	0.761***	0.724***	0.807***

Higher-scoring group	All children (*N* = 26)	Male (*N* = 16)	Female (*N* = 10)
SRS total	0.503**	0.531*	0.498
SCI	0.485**	0.577**	0.375
RRB	0.578**	0.474*	0.788**

Lower-scoring group	All children (*N* = 142)	Male (*N* = 73)	Female (*N* = 69)
SRS total	0.594***	0.574***	0.617***
SCI	0.568***	0.518***	0.639***
RRB	0.607***	0.669***	0.427***

*SRS* Social Responsiveness Scale, *SCI* Social Communication and Interaction, *RRB* Restricted Interests and Repetitive Behavior

***p < 0.001; **p < 0.01; *p < 0.05

In the higher-scoring group, the ICCs for the SRS total, SCI, and RRB scores were moderate for all children (all *p*-values < 0.01). For higher-scoring males, the ICCs of the SRS total, SCI and RRB scores were moderate (*p* < 0.05, < 0.01, < 0.05, respectively). For higher-scoring females, the ICC of the RRB score was substantial (*p* < 0.01), whereas the ICCs of the SRS total and SCI scores were not statistically significant (*p* = 0.052, 0.118, respectively).

In the lower-scoring group, the ICCs for the SRS total and SCI scores were moderate while the ICC of the RRB score was substantial for all children (all *p*-values < 0.001). The same pattern of results was observed in males (all *p*-values < 0.001). In contrast, for females, the ICCs of the SRS total and SCI scores were substantial whereas the ICC of the RRB score was moderate (all *p*-values < 0.001). The mean total, and subdomain scores at age 5 and 8 years of both groups are presented in Table 4.

**Table 4:** SRS raw scores at age 5 and 8 years among the higher- and lower-scoring groups

	Age 5	Age 8	Age 5	Age 8	Age 5	Age 8
	Mean (SD)	Mean (SD)	Mean (SD)	Mean (SD)	Mean (SD)	Mean (SD)
	[Range]	[Range]	[Range]	[Range]	[Range]	[Range]
Higher-scoring group	All children (*N* = 26)		Male (*N* = 16)		Female (*N* = 10)	
SRS total	68.27 (18.83)	63.12 (31.28)	70.13 (17.36)	62.50 (32.52)	65.30 (21.60)	64.10 (30.88)
	[46–117]	[20–132]	[51–111]	[20–132]	[46–117]	[23–113]
SCI	55.46 (14.45)	52.35 (23.84)	56.63 (13.72)	51.88 (24.71)	53.60 (16.13)	53.10 (23.67)
	[38–92]	[19–102]	[38–90]	[19–102]	[40–92]	[21–92]
RRB	12.81 (5.56)	10.77 (8.27)	13.50 (4.94)	10.63 (8.88)	11.70 (6.55)	11.00 (7.65)
	[4–25]	[1–32]	[6–21]	[1–32]	[4–25]	[2–23]

Lower-scoring group	All children (*N* = 142)		Male (*N* = 73)		Female (*N* = 69)	
SRS total	27.05 (10.69)	26.61 (13.66)	27.93 (11.28)	28.47 (15.18)	26.12 (10.01)	24.64 (11.63)
	[5–48]	[4–87]	[5–48]	[4–87]	[5–45]	[4–57]
SCI	23.81 (9.29)	23.47 (11.80)	24.18 (9.59)	24.67 (12.90)	23.42 (9.00)	22.20 (10.46)
	[4–43]	[3–73]	[4–42]	[3–73]	[5–43]	[4–53]
RRB	3.24 (2.41)	3.13 (2.70)	3.75 (2.66)	3.79 (2.98)	2.70 (2.00)	2.43 (2.18)
	[0–12]	[0–14]	[0–12]	[0–14]	[0–9]	[0–9]

*SRS* Social Responsiveness Scale, *SCI* Social Communication and Interaction, *RRB* Restricted Interests and Repetitive Behavior

## Discussion

The aim of the current study was to examine the degree of stability/change in autistic traits during the transition period from preschool to elementary school in a sample of community-based children, and to explore the across-time stability/change for each SCI and RRB subdomain separately, both for males and females and for children with a higher and lower level of autistic traits.

### Main Findings

We found that although autistic traits assessed by the SRS decreased slightly from age 5 to 8, the extent of this change did not reach statistical significance in this sample, indicating that autistic traits are primarily stable during this transition period at the group level. We also found that intra-individual variation in autistic traits appears to be highly preserved between age 5 and 8 which suggests that autistic traits are stable at the individual level during this transition period. To the best of our knowledge, this study is the first to report that autistic traits observed in the general population remain stable during the transition period from preschool to elementary school.

The finding that autistic traits were stable in our participants across the preschool and early school period extends earlier findings among children in the general population who were older than our participants (Constantino et al. 2009; Holmboe et al. 2014; Robinson et al. 2011). The slight decrease observed in mean scores between age 5 and 8 years was not significant in this study, whereas a significant decrease was reported previously for males between ages 7, 10, and 13 (Robinson et al. 2011) and for males and females between age 8 and 12 years (Holmboe et al. 2014). The discordance in these results might be explained by various differences across these studies including cultural differences. For example, as mentioned above, this may relate to the difference in the age range of the children. More specifically, it is possible that autistic traits in the general population might remain primarily stable at a younger age (i.e. 5–8) from preschool to elementary school and decrease during school-age. The highly preserved intra-individual variation in autistic traits observed in this study parallels the findings from previous studies (Constantino et al. 2009; Holmboe et al. 2014; Robinson et al. 2011; Whitehouse et al. 2011).

### Stability of Autistic Traits for Each Subdomain and by Sex

The across-time stability/change in autistic traits was observed for each SCI and RRB subdomain. Autistic traits in males and females remained similarly stable between the ages of 5 and 8 at both the group and individual levels. The finding that there were non-significant mean differences in autistic traits across the study period and a substantial correlation between autistic traits at age 5 and 8 for both the SCI and RRB subdomains, as well as for males and females, respectively is consistent with the results from a study by Holmboe et al. (2014) where three CAST subscale scores (for social impairments, communication impairments, and RRBs and interests) exhibited a high degree of stability in males and females across 8–12 years of age. However, in a study by Whitehouse et al. (2011) of the longitudinal association in autistic traits between age 2 and 19, a significant association was found for total and social autistic traits among males, while for females no significant association was found for either total, social or non-social autistic traits. Despite this interesting difference, it should be noted that the results of our study are not directly comparable with those from the Whitehouse et al. (2011) study because the age interval examined and measures used are different (i.e. they used scales such as the PDP and AQ). Moreover, they also obtained information from different raters (i.e. parents and study subjects themselves) in early childhood and adulthood.

### Sex-Specific Patterns in Children with Higher Autistic Traits

The stability for total autistic traits as well as SCI and RRB subdomain traits was also observed when the sample was divided into higher and lower-scoring groups. This may indicate that during the period between age 5 and 8, irrespective of the degree of severity, autistic traits can be reliably and quantitatively assessed in the general population. However, we found a possible difference in the sex-specific developmental pattern in autistic traits in children with higher autistic traits across preschool and elementary school age. Specifically, for males, intra-individual variation in terms of the SCI and RRB subdomains remained relatively stable, although these scores decreased by 0.4–0.6 SD during the period. In contrast, for females, SCI subdomain autistic traits varied intra-individually a lot over time whereas RRB subdomain autistic traits remained highly stable. On the other hand, their mean SCI subdomain scores were almost the same between age 5 and 8 while (all of) their SRS scores at age 8 were even higher than those of their male counterparts. Considering the small sample size of higher-scoring females, it can be hypothesized that mothers’ judgement concerning their daughters’ social behavior may vary considerably during this period. This suggests that entering elementary school may somehow influence the social, but not repetitive behavior, of females with higher autistic traits, for better or for worse, which is not the case with males. Indeed, recent research has shown that females are more likely than males to experience an escalation in autistic social traits during early- and mid-adolescence based on a large UK birth cohort study (Mandy et al. 2018), which seemingly accords with our finding. Future research with large samples including individuals with well-defined and subthreshold ASD with a wide age range is needed in order to reveal factors that might underlie sex differences in the developmental course and outcomes of autistic traits in the general population.

### Limitations

First, we had no information on the diagnosis of ASD and solely utilized autistic traits as reported by mothers. Second, clinical information such as cognitive and language levels, emotional/behavioral problems, and history of any interventions that could have influenced the change in autistic traits was not obtained. SRS scores may be correlated with IQ in children with IQs < 70, although they are not in children with average IQ (Kamio et al. 2013c). Furthermore, co-occurring anxiety and ADHD symptoms may heighten the SRS scores for children with ASD (Cholemkery et al. 2014; Factor et al. 2017). Third, when interpreting the ICC between two time points, we need to take possible floor effects into consideration, because the scores of some lower-scoring children may be very low. Fourth, families with a low income, less educated parents, and working mothers were under-represented among our participants. Lower maternal education has been associated with an increased risk for autistic traits (Fujiwara 2014) and ASD (Rai et al. 2012), whereas low income has not been associated with autistic traits (Fujiwara 2014) but has with ASD (Rai et al. 2012). In addition, it is possible that mothers with a higher education level and who are full-time housewives may rate their children differently from mothers who are not. Given this, caution should be exercised when it comes to generalizing these results.

## Implications

Given that a substantial proportion of school children with ASD in mainstream school classes may be undiagnosed (Kim et al. 2011), the findings of this study that autistic traits at the age of 5 years can predict autistic traits at age 8 years may have important clinical implications. Specifically, assessing autistic traits before school entrance may aid in predicting later autistic traits as well as other co-occurring social and emotional problems (Saito et al. 2017), and can provide valuable information when it comes to formulating individual educational and mental health care plans at school, especially for children with ASD/subthreshold ASD who are likely to have otherwise gone unrecognized.

## Conclusions

This study demonstrated that autistic traits in a community sample of both males and females are primarily stable in the period between 5 and 8 years of age. This indicates that autistic traits in the general population can be reliably assessed using quantitative measurements for this age band.

## Appendix

**Table:** SRS raw scores of the study participants and of the families who consented to participate in the longitudinal study but who were not included in this study

SRS	All children				Male				Female			
	Participants	Non-participants^a^			Participants	Non-participants^b^			Participants	Non-participants^c^		
	Mean (SD)	Mean (SD)	*t*	*p*	Mean (SD)	Mean (SD)	*t*	*p*	Mean (SD)	Mean (SD)	*t*	*p*
	[Range]	[Range]			[Range]	[Range]			[Range]	[Range]		
Total	33.43 (19.32)	35.13 (17.83)	−0.85	0.39	35.52 (20.52)	36.47 (19.56)	−0.32	0.75	31.08 (17.70)	33.60 (15.58)	−0.97	0.34
	[5–117]	[7–121]			[5–111]	[7–121]			[5–117]	[8–83]		
SCI	28.71 (15.36)	30.14 (14.21)	−0.90	0.37	30.01 (16.26)	31.15 (15.37)	−0.49	0.63	27.24 (14.24)	28.99 (12.75)	−0.82	0.41
	[4–92]	[6–96]			[4–90]	[6–96]			[5–92]	[8–69]		
RRB	4.72 (4.65)	4.99 (4.28)	−0.57	0.57	5.51 (4.91)	5.33 (4.77)	0.25	0.80	3.84 (4.19)	4.61 (3.64)	−13	0.21
	[0–25]	[0–25]			[0–21]	[0–25]			[0–25]	[0–18]		

*SRS* Social Responsiveness Scale, *SCI* Social Communication and Interaction, *RRB* Restricted Interests and Repetitive Behavior

^a^Calculated for 178 participants

^b^Calculated for 95 participants

^c^Calculated for 83 participants
